# The Exact Curve Equation for Majorana Stars

**DOI:** 10.1038/s41598-017-15776-w

**Published:** 2017-11-14

**Authors:** Fei Yao, Dechao Li, Haodi Liu, Libin Fu, Xiaoguang Wang

**Affiliations:** 1 Zhejiang Institute of Modern Physics, Department of Physics, Zhejiang University, Hangzhou, 310027 China; 2 School of Mathematics, Physics and Information Science, Zhejiang Ocean University, Zhoushan, 316022 China; 3 Center for Quantum Sciences and School of Physics, Northeast Normal University, Changchun, 130024 China; 4 National Laboratory of Science and Technology on Computational Physics, Institute of Applied Physics and Computational Mathematics, Beijing, 100088 China

## Abstract

Majorana stars are visual representation for a quantum pure state. For some states, the corresponding majorana stars are located on one curve on the Block sphere. However, it is lack of exact curve equations for them. To find the exact equations, we consider a superposition of two bosonic coherent states with an arbitrary relative phase. We analytically give the curve equation and find that the curve always goes through the North pole on the Block sphere. Furthermore, for the superpositions of SU(1,1) coherent states, we find the same curve equation.

## Introduction

The Majorana representation (MR), in which a pure state of a spin-$j$ system^1,2^ can be precisely described as the trajectory of $2j$ stars on a unit sphere, was proposed by Majorana in 1932^3^. Over the past decades, the MR has been proved to be a valuable method for many applications in quantum entanglement^4–10^, Bose-Einstein condensates^12–16,25^, and geometric phase^10,17–20^. In refs ^10,20^, the Berry phase was studied by the stars and their loops on the Bloch sphere. With the solid angles of the Majorana star loops, an intuitive relation between the Berry phase and Majorana stars’ trajectories on the Bloch sphere was given and it was shown that the Berry phase is determined by both the solid angles subtended by every Majorana star’s evolution path and the correlations between the stars^20^. Quantum entanglement were investigated by distributions and motions of these stars on the Bloch sphere^20^. It is found that the distances between stars are also found to be a tool for measuring and classifying the multiparticle entanglement of a symmetric multiqubit pure state.

The MR was also used to study a multi-band topological systems^21^. They find a geometric interpretation of the topological phases of inversion-symmetric polymerized models by mapping the Bloch states of the topological system to majorana stars. It is interesting to find that the stars displays different topological structures for topologically different phases and the topological structure is closely related to the parity of the system. In addition, it was found that the MR provides a very interesting and intuitive way to understand the nonlinear Laudau-Zener tunneling^22^ and the breakdown of adiabaticity is related to some stars never reaching the South pole of the Bloch sphere.

As an efficient tool to study spin system^23–25^, the MR has certain limitations. It can only be used to study a pure spin-$j$ state in finite dimensional Hilbert space. To solve these problems, a lot of researches^26–30^ concentrate on extending the previous representation. In paper^30^, for instance, Giraud *et al*. extended the MR from pure spin states to arbitrary mixed states. Based on Weinberg’s covariant matrices^31^, they proposed a tensorial representation of the mixed states and expressed any a spin-$j$ density matrix as a linear combination of matrices with convenient properties.

On the other hand, based on the coherent-state approach, the MR was extended from the finite dimensional to infinite dimensional cases^32^. By choosing the coherent states as reference states, the corresponding star equations for locating stars were given for both bosonic and SU(1,1) systems. All the stars for coherent states coincide on one point on the Bloch sphere^32^. Thus, using this coherent-state approach, we can study star representations of many quantum states including both finite and infinite-dimensional systems.

In general, it is hard to give the positions of Majorana stars analytically, and we always locate them numerically. The curve equations for these stars are even harder to be given. Fortunately, we find an exact curve equation for Majorana stars for a superpositon of two bosonic coherent states (STCS) with an arbitrary relative phase. For the superpositions of SU(1,1) coherent states, we find the same curve equation.

In this paper, we give the star equation for the STCS of the bosonic system and deduce the corresponding curve equation for stars on the Bloch sphere, and then explore the curve properties on the Block sphere by the theoretic analysis. Moreover, we discuss the curve properties on the Block sphere with the numerical calculation. Finally, we give our conclusions and some further discussions.

## Results

### Star equation for the STCS and its solution

To find exact curve equation for Majorana stars, we consider the following state, i.e., the STCS,

1 $$|\phi \rangle =|\alpha \rangle +{e}^{i{\vartheta }_{1}}|\beta \rangle ,$$

where ${\vartheta }_{1}$ is the phase difference between two coherent states $|\alpha \rangle$ and $|\beta \rangle$, and the coherent state is given by

2 $$|\alpha \rangle ={e}^{-\frac{{|\alpha |}^{2}}{2}}\sum _{k\mathrm{=0}}^{\infty }\frac{{\alpha }^{k}}{\sqrt{k!}}|k\rangle ,$$

where $\alpha$ is a complex number and $|k\rangle$ is Fock state. The star equation for the STCS is

3 $${e}^{-\frac{{|\alpha |}^{2}}{2}}{(1-\alpha z)}^{N}+{e}^{i{\vartheta }_{1}}{e}^{-\frac{{|\beta |}^{2}}{2}}{(1-\beta z)}^{N}=\mathrm{0,}$$

where $z$ is the root of this star equation. There are $N$ roots ${z}_{n},n=0\,,\,\mathrm{1,}\,2\cdots \cdots N-1$ mapping to the stars on the Block sphere. The equations for the coordinates of the stars can be given as

4 $${\theta }_{n}=2\,\arctan |\frac{1}{\alpha }\frac{R{e}^{i{\gamma }_{n}}-1}{(\lambda R{e}^{i({\gamma }_{n}+{\vartheta }_{2})}-\mathrm{1)}}|$$

5 $$=\,2\,\arctan \,(\frac{1}{|\alpha |}\sqrt{\frac{{R}^{2}+1-2R\,\cos \,{\gamma }_{n}}{{\lambda }^{2}{R}^{2}+1-2\lambda R\,\cos ({\gamma }_{n}+{\vartheta }_{2})}}),$$

6 $${\varphi }_{n}={\rm{\arg }}(R{e}^{i{\gamma }_{n}}-\mathrm{1)}-{\rm{\arg }}(\alpha )-{\rm{\arg }}(\lambda R{e}^{i({\gamma }_{n}+{\vartheta }_{2})}-\mathrm{1).}$$

where

7 $$R={e}^{\frac{{|\alpha |}^{2}-{|\beta |}^{2}}{2N}},$$

8 $${\gamma }_{n}=\frac{{\vartheta }_{1}+\mathrm{(1}+2n)\pi }{N}\mathrm{.}$$

These are the coordinates for all stars on the Block sphere, the detailed calculation can be found in Methods.

On the coordinates for the stars, we now give the further discussions. Firstly, we consider a simple case of $\lambda =\frac{|\beta |}{|\alpha |}=1$, and then $R={e}^{\frac{{|\alpha |}^{2}-{|\beta |}^{2}}{2N}}=1$. So, the Eqs (5) and (6) can be reduced to

9 $${\theta }_{n}=2\,\arctan (\frac{1}{|\alpha |}|\frac{\sin \,\frac{{\gamma }_{n}}{2}}{\sin \,\frac{{\gamma }_{n}+{\vartheta }_{2}}{2}}|),$$

10 $${\varphi }_{n}=-\frac{{\theta }_{\alpha }+{\theta }_{\beta }}{2}\mathrm{.}$$

From above two equations, we know that ${\theta }_{n}$ and ${\varphi }_{n}$ has no relation with ${\vartheta }_{1}$, moreover ${\varphi }_{n}$ is a constant if the total value of ${\theta }_{\alpha }$ and ${\theta }_{\beta }$ is fixed. In this case, the stars’ distributions are independent of the relative phase ${\vartheta }_{1}$ between the two component states. A more particular case is the situation of $\alpha =\beta$. the Eqs (9) and (10) are further reduced to

11 $${\theta }_{n}=2\,\arctan \frac{1}{|\alpha |},$$

12 $${\varphi }_{n}=-{\theta }_{\alpha }.$$

This is what we expected, namely, all the coherent states correspond to one point on the Bloch sphere.

### Exact curve equation for stars

Above we have discussed the case of $|\alpha |=|\beta |$. Now, we discuss the case of $|\alpha |\ne |\beta |$. Based on the Eq. (7), note that $R\approx 1$ when $N\to \infty$. Then, the general spherical coordinates of the stars making up one curve can be written as

13 $${\theta }_{n}=2\arctan (\frac{1}{|{\alpha }|}\sqrt{\frac{2-2\,\cos \,{\gamma }_{n}}{1+{\lambda }^{2}-2\lambda \,\cos ({\gamma }_{n}+{\vartheta }_{2})}}),$$

14 $${\varphi }_{n}=\frac{{\gamma }_{n}}{2}+\frac{\pi }{2}-{\theta }_{\alpha }-{\rm{\arg }}(\lambda {e}^{i({\gamma }_{n}+{\vartheta }_{2})}-\mathrm{1).}$$

These two equations indicate that there is always exist a certain value for ${\gamma }_{n}$, and this certain value always leads to ${\theta }_{n}=0$. It means that the curve of STCS must through the North Pole. This characteristic of the curve will be displayed in figures later. Otherwise, from Eq. (14), we do know the curve which is composed of stars will rotate ${\theta }_{\alpha }$ about $Z$-axis when ${\theta }_{\alpha }\ne 0$.

To further obtain the exact curve equation, we set ${\vartheta }_{2}=\mathrm{0,}\pi$. For ${\vartheta }_{2}=0$, from the Eqs (5) and (6), we can obtain

15 $${\tan }^{{\rm{2}}}\frac{{\theta }_{n}}{2}=\frac{1}{|\alpha {|}^{2}}\frac{{R}^{2}+1-2R\,\cos \,{\gamma }_{n}}{{\lambda }^{2}{R}^{2}+1-2\lambda R\,\cos \,{\gamma }_{n}},$$

16 $${\tan }^{2}\tilde{\varphi }=\frac{{\mathrm{(1}-{\lambda })}^{2}{R}^{2}\mathrm{(1}-{\cos }^{2}{\gamma }_{n})}{{\mathrm{(1}+{\lambda }{R}^{2}-(\lambda +\mathrm{1)}R\cos {\gamma }_{n})}^{2}},$$

where

17 $$\mathop{\varphi }\limits^{ \sim }={\varphi }_{n}+{\theta }_{\alpha }.$$

Because Eqs (15) and (16) are the functions on ${\gamma }_{n}$, we can obtain the relation between ${\tan }^{2}\frac{{\theta }_{n}}{2}$ and ${\tan }^{2}\tilde{\varphi }$ by eliminating ${\gamma }_{n}$. Finally, we obtain

18 $$\begin{array}{c}-y{\mathrm{[1}+x-{R}^{2}\mathrm{(1}+\lambda x)]}^{2}=(x-{\mathrm{1)}}^{2}+{R}^{4}{({\lambda }^{2}x-\mathrm{1)}}^{2}\\ \,\,\,\,-2{R}^{2}\mathrm{\{1}+x\mathrm{[1}+\lambda (\lambda -4+\lambda x)]\}\end{array}$$

where

19 $$x=|\alpha {|}^{2}{\tan }^{2}\frac{{\theta }_{n}}{2},$$

20 $$y={\tan }^{2}{\tilde{\varphi }}_{n}={\tan }^{2}({\varphi }_{n}+{\theta }_{\alpha }\mathrm{).}$$

Equation (18) is the exact equation of the curve. It is only dependent on the polar and azimuth angles and from this equation, all the stars can be determined on the Bloch sphere.

The above we have given the curve equation for $N$, which takes any positive integer. For coherent states, $N\to \infty$, based on the Eq. (7), we obtain $R\approx 1$, since $|\alpha {|}^{2}-|\beta {|}^{2}$ is a finite value. In this case, the Eq. (18) becomes a simpler form

21 $${\tan }^{2}(\frac{\theta }{2})=\frac{4\,{\cos }^{2}(\varphi +{\theta }_{\alpha })}{{|\alpha |}^{2}{(1+\lambda )}^{2}},$$

where ${\lambda }$, ${\theta }_{\alpha }$ and $|\alpha |$ are the constants. This is a curve equation for the superposition of two coherent states. This curve includes infinite stars $({\theta }_{n},{\varphi }_{n})$, *n* = 1, 2, 3, …., ∞ on the Bloch sphere, and we will give the corresponding figures in the next section. Based on the Eq. (21), we know that the curve on the Bloch sphere has no relation with $N$ and the phase difference ${\vartheta }_{1}$ between $|\alpha \rangle$ and $|\beta \rangle$. It means that, when the value of $N$ or ${\vartheta }_{1}$ change, the curve’s shape and location on the Bloch sphere are invariant. Furthermore, combining the Eq. (21) and $\lambda =\frac{|\beta |}{|\alpha |}$, we can obtain

22 $${\tan }^{2}(\frac{\theta }{2})=\frac{4\,{\cos }^{2}(\varphi +{\theta }_{\alpha })}{{(|\alpha |+|\beta |)}^{2}}\mathrm{.}$$

This is the curve equation in the case of no argument difference between $\alpha$ and $\beta$. From this equation, we surely know the curve is invariable as long as ${(|\alpha |+|\beta |)}^{2}$ takes a certain value whatever the values $\alpha$ and $\beta$ respectively take. Meanwhile, because of ${\cos }^{2}(\varphi +{\theta }_{\alpha })\le 1$, this curve is located on the northern hemisphere and through the North Pole in case of ${(|\alpha |+|\beta |)}^{2}\ge 4$. Moreover, the greater the values of ${(|\alpha |+|\beta |)}^{2}$ are, the curve get closer to the North Pole. When ${(|\alpha |+|\beta |)}^{2}\le 4$, the curve runs through the northern and southern hemisphere.

So far, we have discussed the properties of the curve in the case of no argument difference between $\alpha$ and $\beta$. Similarly, the properties of the curve for ${\vartheta }_{2}=\pi$ can be discussed. Using similar derivation, we obtain the curve equation

23 $$\begin{array}{c}-y{\mathrm{[1}+x-{R}^{2}\mathrm{(1}+\lambda x)]}^{2}=(x-{\mathrm{1)}}^{2}+{R}^{4}{({\lambda }^{2}x-\mathrm{1)}}^{2}\\ -2{R}^{2}\mathrm{\{1}+x\mathrm{[1}+\lambda (\lambda +4+\lambda x)]\}\end{array}$$

where $x$, $y$ are the same as Eqs (19) and (20). Moreover, the corresponding curve equation of $N\to \infty$ can be given as

24 $${\tan }^{2}(\frac{\theta }{2})=\frac{4\,{\cos }^{2}(\varphi +{\theta }_{\alpha })}{{(|\alpha |-|\beta |)}^{2}}\mathrm{.}$$

By now, we have derived the curve equation in the case of ${\vartheta }_{2}=\mathrm{0,}\,\pi$ for the STCS. But for other values of ${\vartheta }_{2}$, it is hard to deduce an exact expression of the curve’s equation. Meanwhile, we have to emphasize that a random curve given on the bloch sphere can not be regarded as the expression of the STCS.

### Numerical results of the curve equation

We have presented analytical result for the STCS. In this section, we will further give the numerical results about the STCS in the case of no argument difference (${\vartheta }_{2}=0$) between parameters $\alpha$ and $\beta$.

**Figure 1:**
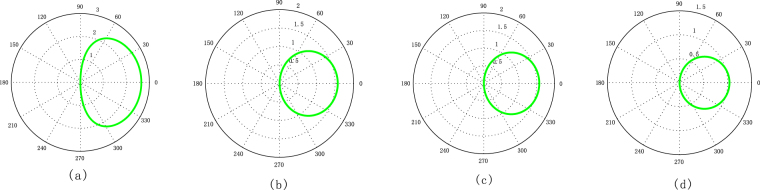
Two-dimensional images for the relation of radius $\theta$ and angle $\varphi$ in Eq. (22) with ${\theta }_{\alpha }={\theta }_{\beta }=0$, (**a**) $|\alpha |=0$, $|\beta |=0.5$, (**b**) $|\alpha |=0.1$, $|\beta |=1.9$, (**c**) $|\alpha |=0.5$, $|\beta |=1.5$, (**d**) $|\alpha |=0.5$, $|\beta |=3$.

In Fig. 1, we give the two-dimensional images about $\theta$ and $\varphi$ with different $|\alpha |$ and $|\beta |$. From the Fig. 1, we know that the curve of STCS through the Northern Pole. And the curve is only located on the northern hemisphere in case of ${|\alpha +\beta |}^{2}\ge 4$, but for ${|\alpha +\beta |}^{2} < 4$, the curve through the northern and southern hemisphere. In addition, comparing Fig. 1-(b) with Fig. 1-(c), the shape and location of the curve is invariant as long as ${|\alpha +\beta |}^{2}$ fixed. Hence the numerical results mentioned above are consistent with the theory in Eq. (22).

**Figure 2:**
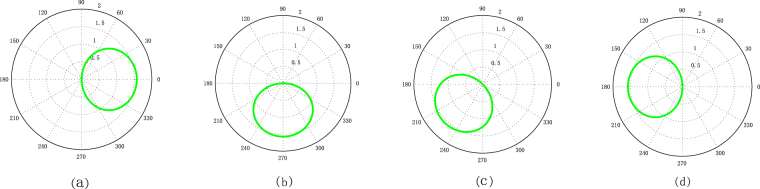
Two-dimensional images for the relation of radius $\theta$ and angle $\phi$ in Eq. (22) with $|\alpha |\,=\,0.5$, $|\beta |\,=\,1.5$, (**a**) ${\theta }_{\alpha }={\theta }_{\beta }\,=\,0$, (**b**) ${\theta }_{\alpha }={\theta }_{\beta }\,=\,0.5\pi$, (**c**) ${\theta }_{\alpha }={\theta }_{\beta }\,=\,0.75\pi$, (**d**) ${\theta }_{\alpha }={\theta }_{\beta }=\pi$.

In Fig. 2, we find that the location of the curve rotate around the northern pole with the increase of ${\theta }_{\alpha }$ and ${\theta }_{\beta }$ while the shape and size is invariant. This numerical result is consistent with the theoretical result in Eq. (22).

In this section, we’ve discussed the impacts on the curve from parameters $\alpha$, $\beta$, ${\theta }_{\alpha }$ and ${\theta }_{\beta }$ with the aid of numerical calculation. And the numerical result is exactly consistent with theoretical result.

## Methods

For a single-mode pure bosonic state

25 $$|\psi \rangle ={e}^{-\frac{{|\alpha |}^{2}}{2}}\sum _{k\mathrm{=0}}^{\infty }\frac{{\alpha }^{k}}{\sqrt{k!}}|k\rangle =\sum _{k\mathrm{=0}}^{\infty }{C}_{k}|k\rangle ,$$

we introduce the state which orthogonal to the state $|\psi \rangle$, i.e.

26 $$\langle z|\psi \rangle =0$$

where

27 $$|z\rangle =\sum _{m\mathrm{=0}}^{N}(\begin{array}{c}N\\ m\end{array})\sqrt{m!}{(-z)}^{m}|m\rangle \mathrm{.}$$

where $N$ is a sufficient large limited cutoff number. Substituting the Eqs (25) and (27) into the Eq. (26), we can obtain the star equation for locating stars as below (The another method of looking for the star equation can be found in^32^.)

28 $$\sum _{k\mathrm{=0}}^{N}\frac{N!}{(N-k)!\sqrt{k!}}{(-1)}^{k}{C}_{k}{z}^{k}=0.$$

For the state given in Eq. (1), the expansion coefficients can be obtained as,

29 $${C}_{k}={e}^{-\frac{{|\alpha |}^{2}}{2}}\frac{{\alpha }^{k}}{\sqrt{k!}}+{e}^{i{\vartheta }_{1}}{e}^{-\frac{{|\beta |}^{2}}{2}}\frac{{\beta }^{k}}{\sqrt{k!}}\mathrm{.}$$

Substituting the above equation into Eq. (28) leads to the following equation for locating stars,

30 $${e}^{-\frac{{|\alpha |}^{2}}{2}}{(1-\alpha z)}^{N}+{e}^{i{\vartheta }_{1}}{e}^{-\frac{{|\beta |}^{2}}{2}}{(1-\beta z)}^{N}=\mathrm{0,}$$

where

31 $$\alpha =|\alpha |{e}^{i{\theta }_{\alpha }},$$

32 $$\beta =|\beta |{e}^{i{\theta }_{\beta }},$$

and $z$ is the root of this star equation. After simplifying, we find out the $n$-th root of $z$ satisfying the relation

33 $$\frac{1-\alpha {z}_{n}}{1-\beta {z}_{n}}=R{e}^{i{\gamma }_{n}},$$

where

$$R={e}^{\frac{{|\alpha |}^{2}-{|\beta |}^{2}}{2N}},{\gamma }_{n}=\frac{{\vartheta }_{1}+\mathrm{(1}+2n)\pi }{N}$$

and $n=\mathrm{0,}\,\mathrm{1,}\,2\cdots \cdots N-1$. Thus, we can obtain the roots of Eq. (30),

34 $${z}_{n}=\frac{1}{{\alpha }}\frac{R{e}^{i{\gamma }_{n}}-1}{(\frac{{\beta }}{\alpha }R{e}^{i{\gamma }_{n}}-1)}$$

35 $$=\frac{1}{\alpha }\frac{R{e}^{i{\gamma }_{n}}-1}{\lambda R{e}^{i({\vartheta }_{2}+{\gamma }_{n})}-1}\mathrm{.}$$

where $\frac{\beta }{\alpha }=\lambda {e}^{i{\vartheta }_{2}}$, $\lambda =\frac{|\beta |}{|\alpha |}$ is a real number, and ${\vartheta }_{2}$ is the argument difference between two coherent parameters $\alpha$ and $\beta$. By now, we have given the roots of star equation for STCS with H-W symmetry. And these roots ${z}_{n}$ can be mapped to the stars on Block sphere via relation

36 $${z}_{n}=\,\tan \,\frac{{\theta }_{n}}{2}{e}^{i{\varphi }_{n}},\,{\theta }_{n}\in [0,\pi ],\,{\varphi }_{n}\in [0,\,2\pi ],$$

where ${\theta }_{n}$ and ${\varphi }_{n}$ are the spherical coordinates. The spherical coordinates can be calculated as Eqs (5) and (6).

## Discussions

In conclusion, for giving curve equation of Majorana stars, we have examined the STCS. For this state, we have obtained exact equations of curve for stars in the case of no argument difference between two parameters of coherent states $\alpha$, $\beta$, or with augment difference $\pi$. These analytic results agree with numerical calculations. Meanwhile, we have shown that the curves through the North Pole. We have further examined the superposition states of two SU(1,1) coherent states, and found the same curve equations. The details are presented in the Appendix. In our investigations, two arbitrary coherent states are superimposed together with equal probability. For the STCS with different probability amplitudes, our method can directly apply.

## Appendix: The curve equation for the STCS of SU(1,1) system

Based on ref.^32^, we know that for the state

37 $$|{\rm{\Phi }}\rangle =|{\zeta }_{1}\rangle +{e}^{i{\tilde{\vartheta }}_{1}}|{\zeta }_{2}\rangle ,$$

where

38 $$|{\zeta }_{1}\rangle ={\mathrm{(1}-|{\zeta }_{1}{|}^{2})}^{k}\sum _{n\mathrm{=0}}^{\infty }{{\zeta }_{1}}^{n}\sqrt{\frac{{\rm{\Gamma }}\mathrm{(2}k+n)}{n!{\rm{\Gamma }}\mathrm{(2}k)}}{|n\rangle }_{k},$$

where Γ is the Gamma-function, and $k$ is Bargmann index,

39 $${|n\rangle }_{k}=\sqrt{\frac{{\rm{\Gamma }}\mathrm{(2}k)}{n!{\rm{\Gamma }}\mathrm{(2}k+n)}}{{K}_{+}}^{n}|0\rangle ,$$

the corresponding star equation is

40 $${\mathrm{(1}-|{\zeta }_{1}{|}^{2})}^{k}{(1-{\zeta }_{1}z)}^{{N}_{c}}+{e}^{i{\tilde{\vartheta }}_{1}}{\mathrm{(1}-|{\zeta }_{2}{|}^{2})}^{k}{\mathrm{(1}-{\zeta }_{2}z)}^{{N}_{c}}=\mathrm{0,}$$

and the ${\zeta }_{1}$, ${\zeta }_{2}$ satisfying

41 $$1-|{\zeta }_{1}{|}^{2}\ne \mathrm{0,}\,1-|{\zeta }_{2}{|}^{2}\ne 0.$$

By similar calculations, we derive the solution of Eq. (40) as follow

42 $${z}_{m}=\frac{\tilde{R}{e}^{i{\gamma }_{m}}-1}{{\zeta }_{1}(\tilde{\lambda }\tilde{R}{e}^{i({\gamma }_{m}+{\tilde{\vartheta }}_{2})}-\mathrm{1)}},$$

where

43 $$\tilde{R}=(\frac{1-|{\zeta }_{2}{|}^{2}}{1-|{\zeta }_{1}{|}^{2}}{)}^{\frac{k}{{N}_{c}}},$$

44 $${\gamma }_{m}=\frac{{\tilde{\vartheta }}_{1}+\mathrm{(1}+2m)\pi }{{N}_{c}},\,m=\mathrm{0,}\,\mathrm{1,}\,\mathrm{...}{N}_{c}-\mathrm{1,}$$

45 $$\tilde{\lambda }=\frac{|{\zeta }_{2}|}{|{\zeta }_{1}|}\mathrm{.}$$

Meanwhile, ${z}_{m}$ can be mapped to the stars on Block sphere via relation in Eq. (36). So, based on the Eqs (36) and (42), we obtain

46 $${\theta }_{m}=2\,\arctan \,(\frac{1}{|{\zeta }_{1}|}\sqrt{\frac{{\tilde{R}}^{2}+1-2\tilde{R}\,\cos \,{\gamma }_{m}}{{\tilde{\lambda }}^{2}{\tilde{R}}^{2}+1-2\tilde{\lambda }\tilde{R}\,\cos \,({\gamma }_{m}+{\tilde{\vartheta }}_{2})}}),$$

47 $${\varphi }_{m}={\rm{\arg }}(\tilde{R}{e}^{i{\gamma }_{m}}-\mathrm{1)}-{\rm{\arg }}({\zeta }_{1})-{\rm{\arg }}(\tilde{\lambda }\tilde{R}{e}^{i({\gamma }_{m}+{\tilde{\vartheta }}_{2})}-\mathrm{1).}$$

These are the coordinates for all stars on the Block sphere. Next, in the same way, we derive the curve equation in ${\theta }_{m}=\mathrm{0,}\pi$

48 $${\tan }^{2}(\frac{\theta }{2})=\frac{4\,{\cos }^{2}(\varphi +{\theta }_{{\zeta }_{1}})}{{(|{\zeta }_{1}|+|{\zeta }_{2}|)}^{2}},{\theta }_{m}=\mathrm{0;}$$

49 $${\tan }^{2}(\frac{\theta }{2})=\frac{4\,{\cos }^{2}(\varphi +{\theta }_{{\zeta }_{1}})}{{(|{\zeta }_{1}|-|{\zeta }_{2}|)}^{2}},{\theta }_{m}=\pi .$$

This is the curve equation for the STCS of SU(1,1) system. Comparing these two equations with the Eqs (22) and (24) for the STCS of bosonic system, we find that they have the same equation form.
